# Laparoscopic Radical Cholecystectomy for Primary or Incidental Early Gallbladder Cancer: The New Rules Governing the Treatment of Gallbladder Cancer

**DOI:** 10.1155/2017/8570502

**Published:** 2017-06-11

**Authors:** Gaetano Piccolo, Guglielmo Niccolò Piozzi

**Affiliations:** ^1^Department of Surgery, University of Catania, Via S. Sofia 78, 95123 Catania, Italy; ^2^Department of Surgery, Università Degli Studi di Milano, Via Festa del Perdono 7, 20122 Milano, Italy

## Abstract

**Aim:**

To evaluate the technical feasibility and oncologic safety of laparoscopic radical cholecystectomy (LRC) for primary or incidental early gallbladder cancer (GBC) treatment.

**Methods:**

Articles reporting LRC for GBC were reviewed from the first case reported in 2010 to 2015 (129 patients). 116 patients had a preoperative diagnosis of gallbladder cancer (primary GBC). 13 patients were incidental cases (IGBC) discovered during or after a laparoscopic cholecystectomy.

**Results:**

The majority of patients who underwent LRC were pT2 (62.7% GBC and 63.6% IGBC). Parenchyma-sparing operation with wedge resection of the gallbladder bed or resection of segments IVb-V were performed principally. Laparoscopic lymphadenectomy was carried out according to the reported depth of neoplasm invasion. Lymph node retrieved ranged from 3 to 21. Some authors performed routine sampling biopsy of the inter-aorto-caval lymph nodes (16b1 station) before the radical treatment. No postoperative mortality was documented. Discharge mean day was POD 5th. 16 patients had post operative morbidities. Bile leakage was the most frequent post-operative complication. 5 y-survival rate ranged from 68.75 to 90.7 months.

**Conclusion:**

Laparoscopy can not be considered as a dogmatic contraindication to GBC but a primary approach for early case (pT1b and pT2) treatment.

## 1. Introduction

Gallbladder cancer (GBC) is the most frequent neoplasm of the biliary tract [1]. GBC has a great worldwide incidence variability in correlation with both geographic and ethnic features.

Higher rates of GBC are observed in South America (especially Chile), Indian subcontinent, Japan, and Korea [2] and, in many cases, this is due to a higher incidence of *S. typhi*/*paratyphi* infection in these countries [3–5].

Nowadays, thanks to the widespread use of ultrasound and laparoscopic cholecystectomy, GBC is diagnosed at an earlier stage with a consequent change in patients' management and outcome.

According to literature, the occurrence of IGBC ranges between 0.19 and 2.8% [6] with almost half of these cases detected after laparoscopic cholecystectomy for benign diseases (polyps, gallstones, and cholecystitis) [7]. IGBC are usually at an earlier pathological stage with consequent increased long-term survival [8, 9].

Simple cholecystectomy may be an adequate treatment only for earlier stages (pTis and pT1a); however, radical resection, including hepatic resection and regional lymphadenectomy, is the only chance of cure associated with demonstrated long-term survival for pT1b and later stages. For many years, the use of laparoscopy in GBC was restricted to staging purposes only.

The most important concerns that led to a preliminary nihilistic approach were as follows: the feasibility of achieving an adequate hepatectomy and lymphadenectomy and the risk of intraoperative peritoneal dissemination with possible port site recurrences.

Recently, few reports have shown the feasibility of laparoscopic radical resection for early gallbladder cancer; however, laparoscopic surgery of the biliary tract still remains a challenging procedure requiring significant experience in both laparoscopy and liver surgery.

## 2. Materials and Methods

### 2.1. Literature Search

MEDLINE and PubMed searches were performed using the key words “laparoscopic treatment of primary gallbladder cancer”, “radical laparoscopic cholecystectomy”, and “incidental gallbladder cancer” in order to identify relevant articles published in literature from the first case reported in 2010 to the last case reported in 2015.

Reference lists from the articles were reviewed to identify additional relevant articles. All studies that contained materials applicable to the topic were considered.

Retrieved manuscripts (case reports and series) were reviewed by the authors, and the data were extracted using a standardized collection tool. The extracted data included general information (number of patients treated, patients' age, and study recruitment period), technical aspects (number of operative instruments used, type of hepatic resection, number of lymph node retrieved, time of operation, and total blood loss), and, if available, oncologic outcomes.

### 2.2. Statistical Analysis

In contrast to classic meta-analyses, the outcome is defined here as the percentages of an event (without comparison) in pseudocohorts of observed patients. Overall proportions can be estimated from the weighted mean of percentages measured in each study. The weight in this case is derived from the number of subjects included in the study out of the total number of subjects in all studies, which is inverse of the variance in the classic meta-analyses.

The confidence interval is calculated through the use of the normal distribution to approximate the binomial probabilities given that the condition “product of the probability and sample size (np) is more than 5” is fulfilled.

## 3. Results

**Figure 1:**
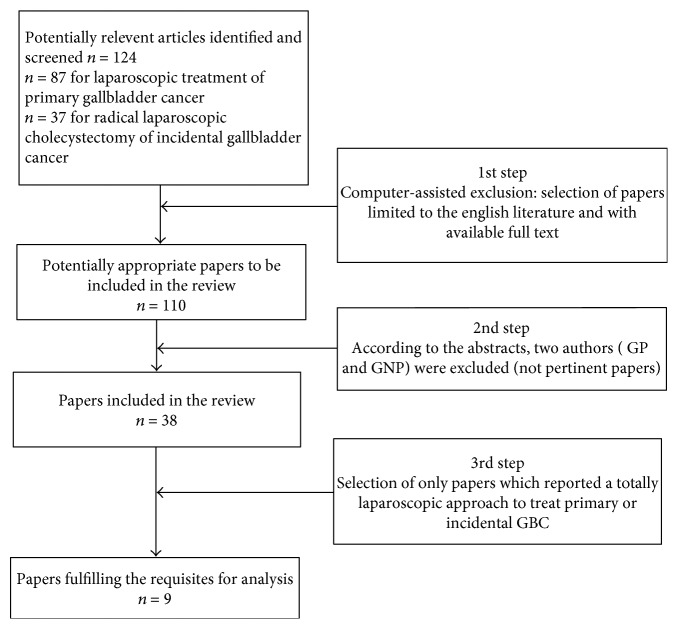
Diagram showing the study methodology and the number of abstracts and articles identified and evaluated during the review process.

Our preliminary review identified 124 papers considered potentially relevant for our analysis. Computer-assisted filtering data allowed to exclude non-English papers and nonavailable full-text articles (*n* = 14). The titles of the 110 retrieved papers were examined by two authors (GP and GNP) who excluded nonpertinent papers. 38 articles were suggestive for our aim, but only 9 articles (including 129 patients) reported a total laparoscopic approach for primary or incidental GBC treatment (Figure 1).

Three articles are case reports while six are retrospective or prospective cohort studies (Table 1). A total of 116 patients had a preoperative suspicion or diagnosis of gallbladder carcinoma, while 13 were incidental gallbladder cancer discovered after a laparoscopic cholecystectomy.

### 3.1. Radical Laparoscopic Cholecystectomy for Primary GBC

At the time of this review, only 7 articles reported a radical laparoscopic approach for primary GBC treatment; among them, one is a case report [10], while the others are retrospective or prospective cohort studies [11–16].

**Figure 2:**
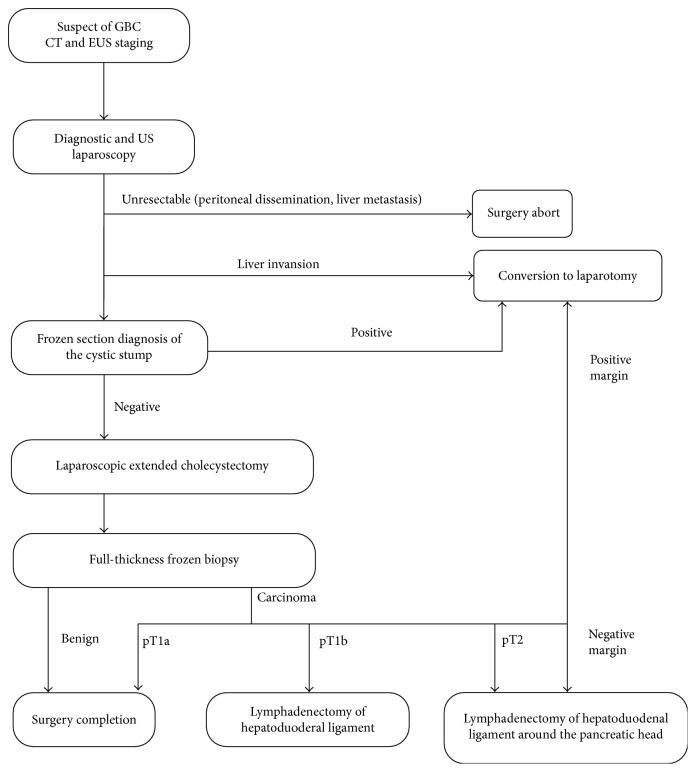
Surgical approach for primary GBC.

In all studies, the majority of patients had a pT2 stage (62.7%) (Table 2). Itano et al. and Yoon et al. [12, 16] reported the two widest prospective cohort studies, including, respectively, 45 and 16 patients with pathologically proven GBC that underwent primary laparoscopic cholecystectomy. Both authors described a similar treatment protocol (Figure 2).

The inclusion criterion was patients with suspected GBC without evidence of liver invasion or extrahepatic bile duct involvement at enhanced abdominal CT scan and preoperative endoscopic ultrasound (EUS) [12, 16]. The endoscopic gallbladder scanning was performed from the bulb or the second portion of the duodenum to the antrum using the balloon contact method.

The EUS staging was used to report the macroscopic tumor appearance (peduncolated versus sessile), the wall thickness (localized versus diffuse), and the layer structures of the gallbladder (maintenance or disruption of the outer hyperechoic layer). All patients were then submitted to laparoscopic staging with both optic and ultrasound analyses in order to exclude unresectable conditions as peritoneal dissemination or liver metastases. In case of liver invasion, the laparoscopic procedure was converted to laparotomy.

Patients with no evidence of liver invasion underwent Calot's triangle dissection with frozen section diagnosis of the cystic duct's stump.

If the biopsy proved to be positive, conversion to laparotomy was performed in order to facilitate a biliary tract reconstruction.

Laparoscopic cholecystectomy was completed by en bloc dissection of a thin liver tissue layer around the gallbladder bed (>1 cm) in order to avoid the risk of bile spillage.

Intraoperative full-thickness frozen biopsy was performed to confirm the depth of tumor invasion.

Laparoscopic lymphadenectomy is then carried out according to the reported depth of the neoplasm invasion [12, 16]. For pT1b cancer (tumor invades muscular layer), regional lymph node dissection (N1 lymph nodes: hilar, cystic, pericholedochal, perihepatic, and periportal lymph nodes) is the best choice of treatment, while for pT2 cancer (tumor invades the perimuscular connective tissue layer), extraregional lymph node dissection (N2 lymph nodes: periduodenal, peripancreatic lymph nodes and lymph nodes around the inferior mesenteric artery, common hepatic, and celiac artery) is recommended [10–19].

Itano et al. proved that the number of dissected lymph nodes during laparoscopic lymphadenectomy was similar to those following the open approach [12].

There were also no statistically significant differences in either the disease-free or overall survival rate between the two approaches.

The landmark of this treatment protocol is the correct determination of the depth of GBC invasion; therefore, EUS, laparoscopic ultrasound (LUS), and intraoperative pathological examination played a fundamental role in optimal treatment strategy [11, 12, 16]. Other authors [14, 15] treated also pT3 stage tumors with routine wedge or segmental hepatic resection of segments IVb and V for pT2 cancers. Resection plane was marked using harmonic hook or monopolar diathermy, and deeper parenchyma division was performed using a combination of harmonic scalpel or LigaSure (Table 3).

### 3.2. Radical Laparoscopic Cholecystectomy for IGBC

Only 13 cases of IGBC are described as full laparoscopically treated in the articles selected in our review. The majority of patients who underwent laparoscopic radical re-resection had a pT2 gallbladder cancer (63.6%) (Table 4).

Shirobe and Maruyama [13] and Agarwal et al. [14] described, respectively, 7 and 4 cases of IGBC treated with laparoscopic radical cholecystectomy, while Machado et al. and Gumbs et al. described only case reports.

Machado et al. reported a case of a 50-year-old woman with a pT1b IGBC who underwent laparoscopic radical re-resection, hepatic resection of segments IVb and V, and laparoscopic extended hilar lymphadenectomy without the need of biliary reconstruction. All 9 lymph nodes retrieved proved to be negative, and the following 12 months follow-up was negative for recurrence.

Gumbs and Hoffman [17] described a pT3 case of IGBC treated with full laparoscopic hepatoduodenal ligament lymphadenectomy and resection of segments IVb and V; the frozen cystic stump margin was proven positive for tumor spread; therefore, a resection of the common bile duct was performed.

A choledochojejunostomy was made using the laparoscopic approach, the Roux limb was then brought up to the common bile duct and anatomized laparoscopically with a single layer of running 4.0 absorbable suture. Also, Shirobe and Maruyama [13] reported two cases of common bile duct resection and biliary tract reconstruction. For this procedure, minilaparotomy was conducted in the first case, while pure laparoscopic approach was performed in the second patient.

Hepatic resection was performed by all authors but with difference in extension [10, 13, 14, 18]. Some authors performed a segmental or a wedge resection of segments IVb and V [13, 14, 17], while Shirobe et al. [18] performed only a 10 mm-wide resection of the gallbladder bed in order to ensure a complete resection of the gallbladder tumor.

The same author considered the hepatic resection not necessary if the tumor was localized on the peritoneum side of the gallbladder.

The liver resection plane is marked, by all authors, using harmonic hook or monopolar diathermy with LUS confirmation of the anatomical landmarks [13, 14, 17, 18]. Liver transection was performed using LigaSure or ultrasonic dissector, while vascular control is enhanced with laparoscopic bipolar device or BiClamp (Table 5).

### 3.3. Laparoscopic Technique

Surgical technique was fully described by all authors. Patient's position was shown by all authors as supine with reverse Trendelenburg and left lateral tilt (low lithotomy or French approach). The operating surgeon stands between the patient's legs while the assistant surgeon on the patient's left. Shirobe and Maruyama [13] described a different approach with the first operator on the right side, the scope operator between the patient's legs, and the assistant on the left side while Palanisamy et al. [15] instead prefers the scope operator on the patient's right side.

The number of port used was 3 for Cho et al. [11] and 4 for Gumbs and Hoffman [17] and Itano et al. [12] while the other authors used 5 [10, 12–16, 18].

The optic port was positioned in the umbilical region while only Gumbs and Hoffman [17] describe a midclavicular positioning below the costal margin. The position of the operative trocars is presented differently by the authors and is showed in Table 6.

A great attention is displayed by all authors in the operative management. The handling of the gallbladder should be minimal, and direct grasping should be avoided in order to reduce the risk of gallbladder rupture with bile spillage and therefore possible tumor cell dissemination.

All authors focus on the need of protected specimen extraction through a plastic bag. The mean length of operation was 276 minutes with a minimum of 90 minutes and a maximum of 441 minutes. The average total blood loss was 210 ml with a minimum of 10 ml and a maximum of 1500 ml. Only one patient in Yoon et al.'s [16] series needed blood transfusion and conversion to laparotomy following portal vein lesion (total intraoperative blood loss: 1500 ml).

Cho et al. [11] reported two intraoperative complications: one patient suffered bleeding from a torn branch of the main portal vein during node dissection that induced conversion to laparotomy; the other case is an injury of the left hepatic duct during LLA treated with intracorporeal repair and T-tube insertion with full postoperative recovery.

### 3.4. Laparoscopic Lymphadenectomy

Before proceeding with the radical resection, Agarwal et al. [14] reported to perform a routine sampling biopsy of the interaortocaval lymph node basins (IAC, 16b1 station), with a median number of 2 lymph nodes analyzed (range: 1–3). Also, Palanisamy et al. [15] looked for enlargement of both IAC and celiac group lymph node basins and executed frozen analysis only if enlarged. Agarwal et al. [14] in case of positive biopsy decided to abandon the surgical resection while Palanisamy et al.'s [15] decision is case-by-case with possible defection of the surgical procedure or additional extension in the nodal clearance.

Lymphadenectomy was performed laparoscopically by all authors, and the mean number of lymph node retrieved ranged between 3 and 21 lymph nodes, according to different authors [10–18].

The extent of lymphadenectomy included lymph nodal dissection along the entire length of the hepatic artery from the celiac axis to its bifurcation into right and left hepatic arteries with dissection of the retropancreatic lymph nodes and lymph nodal clearance of the hepatoduodenal ligament including pericholedochal and peri/retroportal lymph nodes.

The circumferential dissection of the hepatoduodenal ligament is completed, and the entire lymph nodal tissue was excised en bloc. Palanisamy et al. [15] proposed an extended lymphadenectomy with duodenal kocherization.

The duodenum was retracted medially to expose the posterior aspect of the head of the pancreas and continued till exposing the right lateral border of the aorta. All fibrofatty tissues, along the posterior-superior aspect of the pancreatic head, were dissected and swept cranially till the right lateral aspect of the vena cava above the insertion of the right renal vein.

Lymphadenectomy was furtherly executed by entering the lesser sac through the gastrohepatic omentum. The origin of the celiac trunk was exposed with excision of the tissues overlaying the common hepatic artery, safeguarding the gastroduodenal artery.

All the tissues cleared from prior dissected areas were swept toward the hepatoduodenal region in order to be included en bloc in the final specimen.

Finally, the hepatoduodenal ligament was opened, portal structures were skeletonized circumferentially, and lymphadenectomy was completed after removing the periportal, pericholedochal, and the lymph nodes along the hepatic proper artery till its bifurcation. All the resected tissues were then kept in a plastic retrieval bag for removal. The entire fibrofatty tissue was cleared from the cystic triangle skeletonizing the portal branches and the hepatic arteries and swept toward the cystic pedicle to be removed later along with the gallbladder and the liver bed. The lymphadenectomy details are showed in Table 7.

### 3.5. Outcome

None of the series documented postoperative mortality. Discharge mean day was POD 5. An overall 16 patients of the 129 had postoperative morbidities. According to the Clavien-Dindo classification, the classifications are grade I (4 pneumonia, 1 paralitic ileus), grade II (2 voiding difficulty, 2 transient bleeding), and grade IIIa (5 symptomatic intra-abdominal fluid accumulation, 2 wound infection). The most frequent postoperative complication reported was bile leakage.

Palanisamy et al. [15] described postoperative bile leakage in two patients: the first patient underwent ERCP and stenting on POD 5, due to persistent high bilious output; the second reported case was treated through US-guided positioning of percutaneous pigtail catheter. Follow-up mean was 35 months (range: 3–119 months). The 5-year survival rate ranged between 68.75 months and 90.7 months [15, 16].

Palanisamy et al. [15] showed a 5-year survival rate of 68.75% with a median follow-up of 51 months; three patients died during the follow-up period (two had node-positive disease and one had pT3 lesion).

Yoon et al. [16], reporting a prospective 10-year study, showed a median follow-up of 60 months (range: 3.5 to 118.9 months). Two patients deceased for tumor recurrence at 21.3 and 30.3 months after surgery while 4 patients died from newly developed neoplasms (HCC, duct cancer, pancreatic cancer, and gastric cancer; at 40.7, 74.8, 97.2, and 98.3 months after surgery, resp.).

The overall and disease-specific 5-year survival rates for the 45 patients were 90.7% and 94.2%, respectively. The 5-year disease-specific survival rate was 100% for pT1a patients and pT1b patients and 90.2% for pT2 patients.

Shirobe and Maruyama [13] reported a similar finding with a 5-year survival rate of 100% for pT1b patients and 83.3% for pT2 patients.

## 4. Discussion

Gallbladder cancer (GBC) management and outcomes have changed nowadays through worldwide spread of abdominal ultrasound and laparoscopic approach that permits an earlier stage discovery of the disease [6]. For many years, laparoscopic surgery in GBC patients has been contraindicated. Today, GBC and laparoscopy are not two words in contrast. This procedure only does not seem to be a contraindication but also, if correctly performed, may be an elective approach for primary early cases (pT1a, pT1b, and pT2) and a feasible tool for radical re-resection of incidental cases. Survival rate for GBC patients is strictly related to parietal invasion depth of the tumor. Simple cholecystectomy may be an adequate treatment only for earlier stages (pTis and pT1a) [1, 9, 19–22]. Radical cholecystectomy, including hepatic resection with regional lymphadenectomy, is recommended for pT1b and later-stage carcinomas as long as the disease appears to be R0 [1, 9, 19–22]. The primary concerns, which led to a preliminary nihilistic approach to laparoscopy, were the feasibility of achieving an adequate hepatectomy and lymphadenectomy, the risk of intraoperative peritoneal dissemination, and possible port site recurrences.

The mainstay of the radical laparoscopic approach results from a perfect evaluation of the depth of the cancer. This depth assessment may be achieved through endoscopic and laparoscopic ultrasound for primary GBC and through accurate finally pathological examination for incidental cases [12, 16].

In this review, the majority of patients who underwent laparoscopic radical cholecystectomy had a pT2 stage (62.7% and 63.6% for the primary GBC and for the incidental cases, resp.) and only few pT3 stage cases have been treated.

For some authors, tumor invasion into the liver represented a reason to convert the procedure from laparoscopic to open [12, 16]. However, in the era of major liver resections, the wedge gallbladder bed or hepatic resection of segments IVb and V cannot be a concern.

Two aims should be fulfilled during hepatic resection: removal of the tumor that has directly invaded the liver from the gallbladder bed and prevention of micrometastases that may recur around the gallbladder bed.

No consensus is available about the extension of liver resection and whether hepatic resection can prevent liver recurrence.

Nowadays, parenchyma-sparing treatments, such as nonanatomical wedge resection, are preferred to extended ones [10–17].

Wedge resection of the gallbladder bed (3 cm), if hepatoduodenal ligament invasion and locoregional involvement are excluded, is considered preferable to hepatectomy [4, 20].

In case of gallbladder bed invasion, nonanatomical hepatic parenchyma resection with a distal clearance of at least 2 cm is optimal in order to obtain negative histological margins [4, 20].

Lymphadenectomy has an important role in GBC both for staging and as an independent prognostic factor for survival; however, no consensus is available on the lymphadenectomy extension.

Concerns about the accuracy and safety of laparoscopic lymphadenectomy, as the open one, might be related to a surgeon-related variable, dependent mainly on the surgeon's experience and technical skills. For pTis (tumor in situ) and pT1a, simple cholecystectomy, without lymphadenectomy, is considered adequate, although Ogura et al. reported a residual nodal disease in about 2.5% of pT1a [23]. The dissection of the hepatoduodenal ligament-lymph nodes (hilar, cystic, pericholedochal, perihepatic, and periportal lymph nodes) is considered the treatment of choice for pT1b [24].

For pT2 and pT3, extraregional lymph node dissection including periduodenal and peripancreatic lymph nodes and lymph nodes around inferior mesenteric artery, common hepatic, and celiac artery is recommend [19, 25].

Questionable is the choice of performed routine sampling or lymphadenectomy of para-aortic lymph nodes that occurs in approximately 19% of patients with pT2-pT3 GBC [26].

No consensus is available about the prognostic significance of this lymph nodes involvement and if it can be considered a contraindication for radical resection.

According to Murakami et al. [26], no significant difference on overall survival was evidenced among patients with or without metastatic para-aortic lymphatic involvement (*p* = 0.614).

We believe that para-aortic lymph node metastases is not a contraindication for radical resection of gallbladder cancer [27]. Therefore, the positive detection of metastatic para-aortic lymph nodes, during the preliminary pathological examination should not prevent from performing an aggressive surgical procedure and achieving a radical resection [27].

Common bile duct excision and choledochojejunostomy, necessary in case of cystic duct infiltration, are not an absolute contraindication for the laparoscopic approach; however, high technical skills and laparoscopic experience are needed [17].

Port site metastasis is the most common form of peritoneal dissemination after laparoscopic cholecystectomy for GBC and represents the major concern for a laparoscopic approach among surgeons. The prevalence of tumor seeding in port sites is very variable in published series (between 0 and 40%), with higher incidences associated with gallbladder perforation at the time of cholecystectomy [28, 29].

It has been reported at all stages of GBC and at any of the trocars sites. Port site metastasis appears after latency, between few months and 4 years, implying that subclinical port site disease may be unrecognized if excision is not performed.

However, in a large cohort of patients, from the French registry and the Memorial Sloan Kettering Cancer Center (MSKCC), the authors concluded that port site excision was not associated with improved survival and should not be considered mandatory during definitive surgical treatment of incidental gallbladder cancer [29, 30].

## 5. Conclusions

Our study is subjected to a number of limitations, the most important of which is the relatively small group of patients with primary or incidental GBC treated with a total laparoscopic approach. However, in the era of mini-invasive surgery, the rules governing the treatment of GBC are fundamentally changed and laparoscopy does not seem to be not only a contraindication but also, if correctly performed, may be an elective primary approach for the treatment of early cases. The limits of laparoscopy technique are continually redefined by going beyond them every day. Today, laparoscopic approach could be offered to all patients with early resectable disease (pT1b and pT2 cancer).

## Figures and Tables

**Table 1:** Studies.

Author	Date of study	Type of publication	Number of patients	Number of primary GBC	Number of incidental GBC
Cho et al. [11]	2010	Retrospective comparative study	18	18	—
Gumbs and Hoffman [17]	2010	Case report	1	—	1
Gumbs and Hoffman [10]	2010	Case report	1	1	—
Itano et al. [12]	2007–2013	Prospective comparative study	16	16	—
Shirobe and Maruyama [13]	2001–2013	Retrospective study	11	4	7
Agarwal et al. [14]	2011–2013	Retrospective comparative study	24	20	4
Palanisamy et al. [15]	2008–2013	Retrospective study	12	12	—
Machado et al. [18]	2015	Case report	1	—	1
Yoon et al. [16]	2004–2014	Prospective cohort study	45	45	—

**Table 2:** Primary GBC staging.

Author	Cho et al. [11]	Gumbs and Hoffman [10]	Itano et al. [12]	Shirobe and Maruyama [13]	Agarwal et al. [14]	Palanisamy et al. [15]	Yoon et al. [16], 2015	Weighted average, % (95% CI)
	*n*/total (%)	*n*/otal (%)	*n*/total (%)	*n*/total (%)	*n*/total (%)	*n*/total (%)	*n*/total (%)	
pTis	2/18 (11%)	—	—	—	—	—	2/45 (4.4%)	6.3% (11–1.6)
pT1a	2/18 (11%)	—	1/16 (6.25%)	—	—	—	10/45 (22.2%)	16.4% (7.6–25.2)
pT1b	4/18 (22%)	1/1 (100%)	2/16 (12.5%)	2/4 (50%)	1/20 (5%)	—	8/45 (17.8%)	17.3% (10–24.5)
pT2	10/18 (56%)	—	13/16 (81.25%)	2/4 (50%)	11/20 (55%)	11/12 (91.6%)	25/45 (55.5%)	62.7% (59.5–65.8)
pT3	—	—	—	—	8/20 (40%)	1/12 (8.3%)	—	28.1% (24.4–31.8)

**Table 3:** Primary GBC surgery details.

Author	Liver resection	Devices for liver parenchymal transaction	Cystic duct infiltration	Common bile duct resection
Cho et al. [11]	Wedge resection of the gallbladder bed (2 mm)	—	No	No
Gumbs and Hoffman [10]	Segmental resection of IVb and V	Harmonic scalpel	No	No
Itano et al. [12]	Wedge resection of the gallbladder bed (>1 cm)	Harmonic scalpel, LigaSure	No	No
Shirobe and Maruyama [13]	Wedge resection of the gallbladder bed (1 cm)	Ultrasonic coagulating shear, BiClamp	No	No
Agarwal et al. [14]	Wedge resection of segments IVb and V	Harmonic scalpel, LigaSure, ultrasonic aspirator (CUSA)	No	No
Palanisamy et al. [15]	Segmental resection of IVb and V	Harmonic scalpel, LigaSure, bipolar diathermy	No	No
Yoon et al. [16]	Wedge resection of the gallbladder bed (>1 cm)	—	No	No

**Table 4:** IGBC staging.

Study	Gumbs and Hoffman [17]	Shirobe and Maruyama [13]	Agarwal et al. [14]	Machado et al. [18]	Weighted average, % (95% CI)
	*n*/total (%)	*n*/total (%)	*n*/total (%)	*n*/total (%)	
pT1a	—	—	—	—	—
pT1b	—	1/7 (14.3%)	2/4 (50%)	1/1 (100%)	33.3% (30.4–36.3)
pT2	—	6/7 (85.7%)	1/4 (25%)	—	63.6% (62.1–5.2)
pT3	1/1 (100%)	—	1/4 (25%)	—	40% (37.1–42.9)

**Table 5:** IGBC surgery details.

Author	Liver resection	Devices for liver parenchymal transaction	Cystic duct infiltration	Common bile duct resection
Gumbs and Hoffman [17]	Segmental resection of IVb and V	Harmonic scalpel	Yes	Yes
Shirobe and Maruyama [13]	Wedge resection of the gallbladder bed (1 cm)	Ultrasonic coagulating shear, BiClamp	Yes for 2/7 patients	Yes in 2 patients
Agarwal et al. [14]	Wedge resection of segments IVb and V	Harmonic scalpel, LigaSure, ultrasonic aspirator (CUSA)	No	No
Machado et al. [18]	Segmental resection of IVb and V	Harmonic scalpel, LigaSure, bipolar diathermy	No	No

**Table 6:** Laparoscopic technique details.

Author	Position	Number of ports	Optic trocar	1st operative trocar	2nd operative trocar	Assistant trocar	Other trocars	Extraction site	Intraoperatory US
Cho et al. [11]	—	4	Umbilical	—	—	—	—	Umbilical	Yes
Gumbs and Hoffman [17]	French	4	10 mm—right midclavicular below costal margin	12 mm—umbilical	12 mm—right axillary line	5 mm—left upper quadrant	—	—	Yes
Gumbs and Hoffman [10]	French	4	10 mm—right midclavicular below costal margin	12 mm—umbilical (?)	12 mm—right axillary line	5 mm—left upper quadrant	—	Extended umbilical incision	Yes
Itano et al. [12]	French	4	15 mm—umbilical	12 mm—upper medial abdomen	5 mm—right subcostal area right subcostal	5 mm—left subcostal	—	—	Yes
Shirobe and Maruyama [13]	French	5	11 mm—above umbilicus	12 mm—left midclavicular line	12 mm—midclavicular line right	5 mm—subxiphoid	5 mm—right anterior axillary line	—	—
Agarwal et al. [14]	French	5	Infraumbilical	11 mm—left pararectal port (above umbilicus)	5 mm—right pararectal	5 mm epigastric	5 mm—left midclavicular	Periumbilical incision	—
Palanisamy et al. [15]	French	5	10 mm—supra umbilical	10 mm—left subcostal space midclavicular line	5 mm—left subcostal space midclavicular line	10 mm—epigastric	5 mm—right anterior axillary line	Suprapubic incision	Yes
Machado et al. [18]	French	4	10 mm—right midclavicular 3 cm above umbilicus	12 mm—umbilicus	5 mm—right axillary line	5 mm—subxiphoid		Extended umbilical incision	—
Yoon et al. [16]	French	4-5	/	/	/	/		Umbilical	Yes

**Table 7:** Lymphadenectomy.

Author	IAC sampling biopsy	Station of lymph nodes	Number of lymph nodes retrieved
Cho et al. [11]	—	Pericholedochal, hilar, periportal, and common hepatic	8 (4–21)
Gumbs and Hoffman [17]	—	Hepatoduodenal	3
Gumbs and Hoffman [10]	—	Hepatoduodenal	6
Itano et al. [12]	—	Hepatoduodenal ligament (if pT1b); hepatoduodenal ligament and peripancreatic (if pT2)	12.6 ± 3.1
Shirobe and Maruyama [13]	—	Celiac axis, common hepatic, proper hepatic, hepatoduodenal ligament, posterior surface of the pancreas	13.3 ± 2.3
Agarwal et al. [14]	2 (1–3)	Celiac axis, hepatoduodenal ligament (pericholedochal and peri/retroportal included), common hepatic artery, retropancreatic	12.5 ± 5.4 (Primary GBC: 13.6 ± 4.8) (IGBC: 5.5 ± 1.7)
Palanisamy et al. [15]	IAC and celiac (if enlarged)	Posterior surface of the pancreas head and right lateral vena cava; celiac axis, hepatoduodenal ligament, common hepatic artery	8 (4–14)
Machado et al. [18]	—	Extensive hilar and hepatoduodenal ligament	9
Yoon et al. [16]	—	Hepatoduodenal ligament, common hepatic artery	7 (1–15)

## Additional Points

*Core Tip*. This study aims to evaluate the technical feasibility and the oncological safety of laparoscopic radical cholecystectomy (LRC) for primary or incidental early gallbladder cancer (GBC) treatment. In the minimally invasive surgery era, the rules governing the treatment of gallbladder cancer are changing, with laparoscopy not anymore considered a contraindication but, if correctly performed, an elective primary approach for treatment of early cases.
